# The Montreal cognitive assessment: normative data from a large, population-based sample of Chinese healthy adults and validation for detecting vascular cognitive impairment

**DOI:** 10.3389/fnins.2024.1455129

**Published:** 2024-07-31

**Authors:** Qiang Wei, Baogen Du, Yuanyuan Liu, Shanshan Cao, Shanshan Yin, Ying Zhang, Rong Ye, Tongjian Bai, Xingqi Wu, Yanghua Tian, Panpan Hu, Kai Wang

**Affiliations:** ^1^Department of Neurology, The First Affiliated Hospital of Anhui Medical University, Hefei, China; ^2^Anhui Province Key Laboratory of Cognition and Neuropsychiatric Disorders, Hefei, China; ^3^Collaborative Innovation Center of Neuropsychiatric Disorders and Mental Health, Hefei, China; ^4^Wuhan WuDong Hospital, Wuhan, China; ^5^The School of Mental Health and Psychological Sciences, Anhui Medical University, Hefei, China; ^6^Department of Psychology and Sleep Medicine, The Second Affiliated Hospital of Anhui Medical University, Hefei, China; ^7^Institute of Artificial Intelligence, Hefei Comprehensive National Science Center, Hefei, China

**Keywords:** Montreal cognitive assessment, healthy adults, normative data, vascular cognitive impairment, cut-off value

## Abstract

**Background:**

The Montreal Cognitive Assessment (MoCA) is a valuable tool for detecting cognitive impairment, widely used in many countries. However, there is still a lack of large sample normative data and whose cut-off values for detecting cognitive impairment is considerable controversy.

**Methods:**

The assessment conducted in this study utilizes the MoCA scale, specifically employing the Mandarin-8.1 version. This study recruited a total of 3,097 healthy adults aged over 20 years. We performed multiple linear regression analysis, incorporating age, gender, and education level as predictor variables, to examine their associations with the MoCA total score and subdomain scores. Subsequently, we established normative values stratified by age and education level. Finally, we included 242 patients with vascular cognitive impairment (VCI) and 137 controls with normal cognition, and determined the optimal cut-off value of VCI through ROC curves.

**Results:**

The participants in this study exhibit a balanced gender distribution, with an average age of 54.46 years (SD = 14.38) and an average education period of 9.49 years (SD = 4.61). The study population demonstrates an average MoCA score of 23.25 points (SD = 4.82). The multiple linear regression analysis indicates that MoCA total score is influenced by age and education level, collectively accounting for 46.8% of the total variance. Higher age and lower education level are correlated with lower MoCA total scores. A score of 22 is the optimal cut-off value for diagnosing vascular cognitive impairment (VCI).

**Conclusion:**

This study offered normative MoCA values specific to the Chinese adults. Furthermore, this study indicated that a score of 26 may not represent the most optimal cut-off value for VCI. And for detecting VCI, a score of 22 may be a better cut-off value.

## Background

Age-related cognitive impairment represents a significant public health and social challenge in the contemporary era. In 2018 alone, approximately 50 million individuals worldwide were affected by this condition, with projections estimating that the number will triple by 2050, leading to a staggering economic loss of nearly $4 trillion (Rundek et al., 2022). Cerebrovascular pathology is the most important factor in cognitive impairment and has an additional or synergistic effect with neurodegenerative pathology (Rundek et al., 2022). Vascular cognitive impairment (VCI) includes a series of varying degrees of cognitive impairment, ranging from mild cognitive impairment to vascular dementia caused by cerebral ischemic (or hemorrhagic) etiology or vascular factors alone or in combination with neurodegenerative changes (Zhang et al., 2019). VCI accounts for approximately 20–40% of all dementia patients (Wolf, 2012). For cognitive disorders, early identification is of paramount importance to maximize the effectiveness of interventions, which may include counseling, psychoeducation, cognitive training, and medication (De Roeck et al., 2016). Owing to the absence of effective treatments for advanced dementia, the significance of early diagnosis and intervention during the mild cognitive impairment (MCI) stage has gained widespread recognition as a pivotal approach in disease management. These strategies hold the potential to impact long-term outcomes (Zhuang et al., 2021). The early detection and identification of cognitive decline necessitates a straightforward, easily comprehensible, and highly diagnostic tool. Historically, the Mini-Mental Status Examination (MMSE) served as the widely adopted assessment tool (Folstein et al., 1975). Nevertheless, owing to the absence of executive function assessment (Fu et al., 2017), the MMSE demonstrates limited sensitivity in identifying mild cognitive impairment (Roalf et al., 2013; Ciesielska et al., 2016). Subsequently, the Montreal Cognitive Assessment (MoCA) emerged as a viable alternative (Nasreddine et al., 2005), a widely utilized international neuropsychological screening tool, proficient in evaluating a subject’s global cognitive function. In comparison to the MMSE, MoCA incorporates additional tasks to assess visuospatial abilities, as well as specific evaluations of executive function, attention and delayed recall (Smith et al., 2007; Dong et al., 2010). Consequently, it exhibits heightened sensitivity and specificity in detecting cognitive impairment, particularly mild cognitive impairment (Damian et al., 2011; Oudman et al., 2014), and it has been widely recognized and widely used in clinical and scientific research.

The MoCA scale employs cognitive tasks that are rapid, sensitive, and easily manageable, and it has undergone multiple revisions throughout its usage. Over the past decade, the scale has undergone translation into multiple languages and validation in diverse populations encompassing various ages and educational backgrounds (Kenny et al., 2013; Narazaki et al., 2013; Conti et al., 2015; Malek-Ahmadi et al., 2015; Santangelo et al., 2015; Konstantopoulos et al., 2016; Larouche et al., 2016; Borland et al., 2017; Kopecek et al., 2017; Rossetti et al., 2017; Apolinario et al., 2018; Thomann et al., 2018; Cesar et al., 2019; Hayek et al., 2020; Serrano et al., 2020; Davis et al., 2021; Muayqil et al., 2021; Classon et al., 2022; Gagnon et al., 2022; Gaete et al., 2023). However, owing to variations in cultural and linguistic practices across different countries and regions, the performance of MoCA may exhibit variability. As a result, it is particularly important to make reasonable localization revisions to the MoCA scale and develop appropriate local normative values, this approach has been implemented in numerous countries (Rossetti et al., 2011; Narazaki et al., 2013; Conti et al., 2015; Larouche et al., 2016; Borland et al., 2017; Kopecek et al., 2017; Rossetti et al., 2017; Apolinario et al., 2018; Thomann et al., 2018; Cesar et al., 2019; Hayek et al., 2020; Muayqil et al., 2021; Classon et al., 2022; Gaete et al., 2023). At present, MoCA has multiple Chinese versions, including Beijing, Changsha, Cantonese, Hong Kong, Taiwan, and Mandarin version.^1^ However, it must be mentioned that the MoCA has been updated to version 8.1, but there has been a lack of matching Chinese Mandarin version scales, as well as a lack of matching large sample based normative data for the Chinese population. To address this gap, this study used Mandarin-8.1 version (Chinese Mandarin version), which was localized and revised based on the language and cultural characteristics of the Chinese population, some tests, such as alternating connection (“A,” “B,” “C,” “D,” and “E” are adjusted to the “one,” “two,” “three,” “four,” and “five” of Chinese characters) and memory (“Velvet” and “Church” adjusted to “Silk” and “School”), were adjusted to common Chinese characters, while the sentence repetition part was not only adjusted to common words, but also modified the expression of sentences (see footnote 1) Therefore, the main objective of this study is to recruit a large, geographically diverse, and population-based sample based on a higher quality Mandarin-8.1 version of the MoCA scale, in order to develop normative MoCA data stratified by age and education level that is suitable for the Chinese population.

Over an extended period of MoCA implementation, scores below 26 out of 30 were deemed indicative of cognitive impairment; however, empirical investigations have established that despite its high sensitivity, this threshold exhibits limited specificity (Carson et al., 2018; Davis et al., 2021), so it may not be applicable to people with VCI (Pendlebury et al., 2012). At the same time, studies have shown that a slightly lower cut-off value seems to improve the overall testing properties of VCI compared to a score of 26 (Pendlebury et al., 2013; Lees et al., 2014). Overall, it seems meaningful to further clarify more appropriate cut-off values. Therefore, the secondary objective is to further validate the effectiveness of the Mandarin-8.1 version of the MoCA scale in the Chinese population with VCI and explore optimal cut-off value for detecting VCI in the Chinese population.

## Materials and methods

### Study design and population

This study comprised a total of 3,097 cognitively healthy adults from various provinces, autonomous regions, and municipalities directly under the Central Government of Mainland China. Simultaneously include 242 patients with VCI and 137 controls without cognitive impairment who are matched with general conditions.

Participants who meet the following eligibility criteria can undergo MoCA assessment. The participants of cognitively healthy individuals met the following inclusion criteria: (1) age of 20 years or older, (2) proficiency in speaking and understanding Mandarin Chinese, (3) provision of informed consent, (4) capability to effectively complete the MoCA scale, (5) ability to take care of themselves in daily life, and (6) no complaints of cognitive impairment. Exclusion criteria encompassed: (1) presence of dementia, mild cognitive impairment, or any known cognitive impairment attributed to neurological or non-neurological disorders or treatments (e.g., chemotherapy), (2) history of stroke within the past 5 years, (3) manifestation of moderate or severe mental disorders, and (4) presence of other conditions precluding successful completion of the MoCA test.

The participants of patients with or without VCI met the following inclusion criteria (1) age of 40 years or older, (2) proficiency in speaking and understanding Mandarin Chinese, (3) provision of informed consent, (4) capability to effectively complete a battery of neuropsychological tests, (5) the Hachinski scores above 7, and (6) two neuropsychologists assessed that it meets the definition of The Vascular Impairment of Cognition Classification Consensus Study (VICCCS) (Rundek et al., 2022). Exclusion criteria encompassed: (1) There are other diseases and factors that affect cognitive function, such as brain tumors, normal intracranial pressure hydrocephalus, depression, mental illness, and Alzheimer’s disease, Parkinson’s disease, brain trauma caused by drug abuse; (2) Severe visual, auditory, language, and physical impairments, and inability to complete corresponding test scales; (3) Illiteracy, family members refusing to evaluate with a complete set of neuropsychological scales, or lack of cooperation in the evaluation process; (4) Contraindications to MRI or factors that affect imaging quality, such as pacemakers, cochlear implants, metal dentures, etc.; (5) History of head and neck stent implantation, balloon dilation, carotid endarterectomy, aneurysm embolization, arteriovenous malformation embolization, or open head and neck surgery.

Diagnosis of VCI is as follows: (1) Cognitive impairment is defined as: a set of neuropsychological tests in which one or more cognitive dimensions score below the mean by 1.5 standard deviation or above (Chen et al., 2016; Huang et al., 2018); (2) MRI examination clearly shows cerebrovascular pathological changes (white matter hyperintensities (WMH) or ischemic stroke patients); (3) Cognitive impairment is causally related to cerebrovascular pathological changes; (4) The Hachinski scores above 7 (Johnson et al., 2014).

All participants provided written informed consent was obtained in accordance with the Declaration of Helsinki, and this study was approved by the Anhui Medical University Ethics Committee.

### Neuropsychological assessment

#### Translation and cultural modification of the MoCA

Compared with the original English version of MoCA 8.1, the Mandarin-8.1 version has been localized and revised based on the language characteristics and cultural habits of the Chinese population, mainly manifested in the vocabulary used in alternating connection tests, memory tests, attention tests, sentence repetition tests, and word fluency tests (see footnote 1). The total score comprises 30 points, and the assessment can be completed in approximately 10 min. The scoring is divided into various cognitive domains, including visual space/executive function (0–5 points), naming function (0–3 points), attention function (0–6 points), language function (0–3 points), abstraction function (0–2 points), delayed recall function (0–5 points), and orientation function (0–6 points).

Besides the cognitive screening tests of MoCA (Mandarin-8.1 version), all the participants in detecting VCI cut-off values underwent a battery of standardized neuropsychological tests of memory, language, attention, executive function, and visuospatial ability. The tests included: memory (Auditory Verbal Learning Test [AVLT]-immediate recall, delayed recall, and recognition) (Maj et al., 1994), language (Verbal Fluency Test-categories of animals, fruits, and vegetables) (Mok et al., 2004), attention (Digit Span Test [DST] forward and Color Trails Test [CTT]-1) (Dai et al., 1990), executive function (Stroop Color Word Test [SCWT]-dot, word, colored word, Color Trails Test [CTT]-2 and DST backward) (Dai et al., 1990; Lee and Chan, 2000; Cong et al., 2023), and visuospatial ability (Clock Drawing Test) (Sunderland et al., 1989).

All recruiters and evaluators have received training from neurologists well-versed in Neuropsychology testing methodologies.

### Statistical analysis

This study used SPSS 20.0 for statistical analysis, and multiple linear regression methods were used to analyze the relationship between age, gender, education level, MoCA total score, and various sub cognitive domain scores. To obtain reliable normative values, age was categorized into six groups (<40 years old, 40–49 years old, 50–59 years old, 60–69 years old, 70–79 years old, and ≥ 80 years old), while education level was classified into four groups (illiteracy, primary school, middle school, and high school and above). We also used receiver operating characteristic (ROC) curve analysis for sensitivity and specificity analysis. The comparison of ROC curves was performed using Hanley and McNeil’s (1983) area under the curve (AUC) method (0.5 ≤ AUC <0.7, no apparent accuracy, 0.7 ≤ *f* < 0.8, modal accuracy, 0.8 ≤ *f* < 1, good accuracy) (Hanley and McNeil, 1983; Huang et al., 2023). Based on research data, the corresponding score that produces the highest Youden’s Index was selected as the optimal cut-off value. *p* < 0.05 indicates a significant statistical difference.

## Results

### Demographic information of normative data

Among the 3,097 participants in this study, 1,595 were male (approximately 51.50%). The average age of the sample is 54.46 years (SD = 14.38), and the average educational is 9.49 years (SD = 4.61). The specific distribution can be seen in Table 1.

**Table 1:** Demographics distribution of 3,097 participants.

Age group, y	Education level									
	Illiteracy		Primary school		Middle school		High school and above		Total by age	
	M	F	M	F	M	F	M	F	M	F
<40	–	–	–	–	29	24	163	196	192	220
40–49	5	19	36	60	106	118	157	140	304	337
50–59	5	24	94	143	200	148	194	163	493	478
60–69	18	41	90	60	87	59	133	82	328	242
70–79	19	49	65	52	50	24	57	38	191	163
≥80	10	18	41	20	19	11	17	13	87	62
Total by education	57	151	326	335	491	384	721	632	1,595	1,502

M, Male; F, Female; y, years.

### Demographic influences on the MoCA score

**Figure 1:**
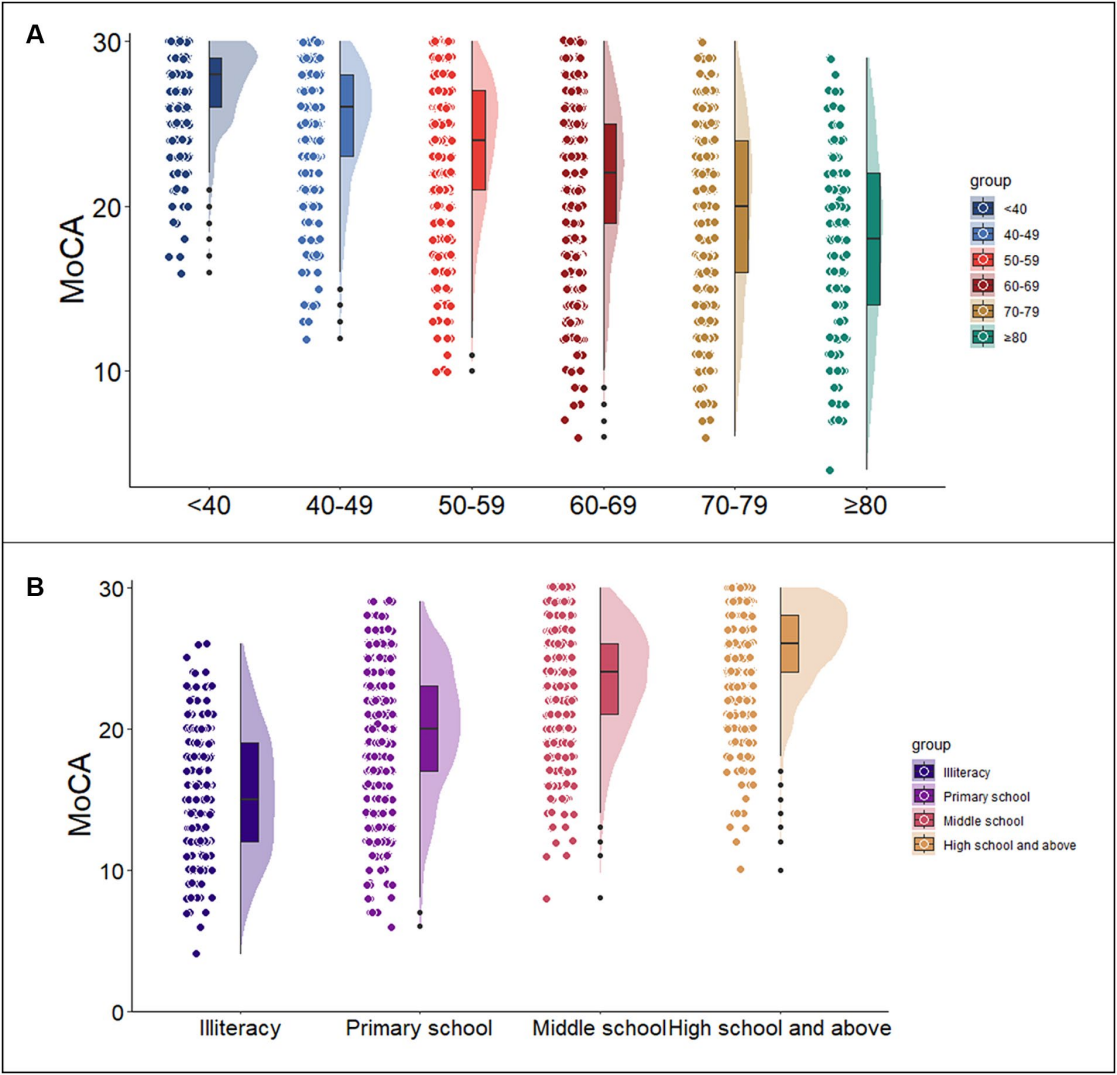
Association of the MoCA total score with age and education. Figures **(A,B)** show the trends of MoCA scores with age group and education level, respectively. (MoCA = 23.900–0.097 * Age+0.522 * Education). MoCA, Montreal Cognitive Assessment.

Multivariate linear regression analysis was used to assess the impact of demographic information on the MoCA total score and subdomain scores. The regression model incorporating age and education demonstrated the highest predictive capability for the MoCA total score (MoCA = 23.900–0.097*Age+0.522*Education, adjusted R^2^ = 0.468, *F* = 907.970, *p*<0.05), explaining 46.8% of the variance. In the regression analysis, increasing age (*p* < 0.05), less education (*p* < 0.05) were associated with a lower MoCA total score, as shown in Table 2 and Figure 1. It was also found that age, education, and gender predicted the scores of each sub cognitive domain to varying degrees. The detailed results of multiple linear regression analysis can be seen in Table 2. In addition, Supplementary Figure S1 shows the standard regression coefficients for predicting the MoCA total score and subdomain scores based on demographic factors.

**Table 2:** Multiple linear regression and beta unstandardized coefficients for the MoCA scores.

Cognitive domains	R	R^2^	F	P	Beta (unstandardized coefficients)		
					Age	Gender	Education
MoCA total score	0.684	0.468	907.970	<0.05	−0.097^*^	−0.211^+^	0.522^*^
Domain subscores							
Visuospatial ability & Executive function	0.587	0.345	543.126	<0.05	−0.016^*^	−0.141^*^	0.148^*^
Naming	0.416	0.173	216.238	<0.05	−0.012^*^	−0.161^*^	0.039^*^
Attention	0.485	0.235	316.930	<0.05	−0.013^*^	−0.096^*^	0.080^*^
Language	0.458	0.210	274.067	<0.05	−0.011^*^	0.039^+^	0.065^*^
Abstraction	0.492	0.242	328.615	<0.05	−0.007^*^	−0.085^*^	0.071^*^
Delayed recall	0.450	0.202	261.553	<0.05	−0.034^*^	0.243^*^	0.080^*^
Orientation	0.270	0.073	81.092	<0.05	−0.004^*^	−0.010^+^	0.040^*^

^*^Represents a significance level less than 0.05, ^+^represents a significance level greater than 0.05.MoCA, Montreal Cognitive Assessment.

### MoCA total score

**Figure 2:**
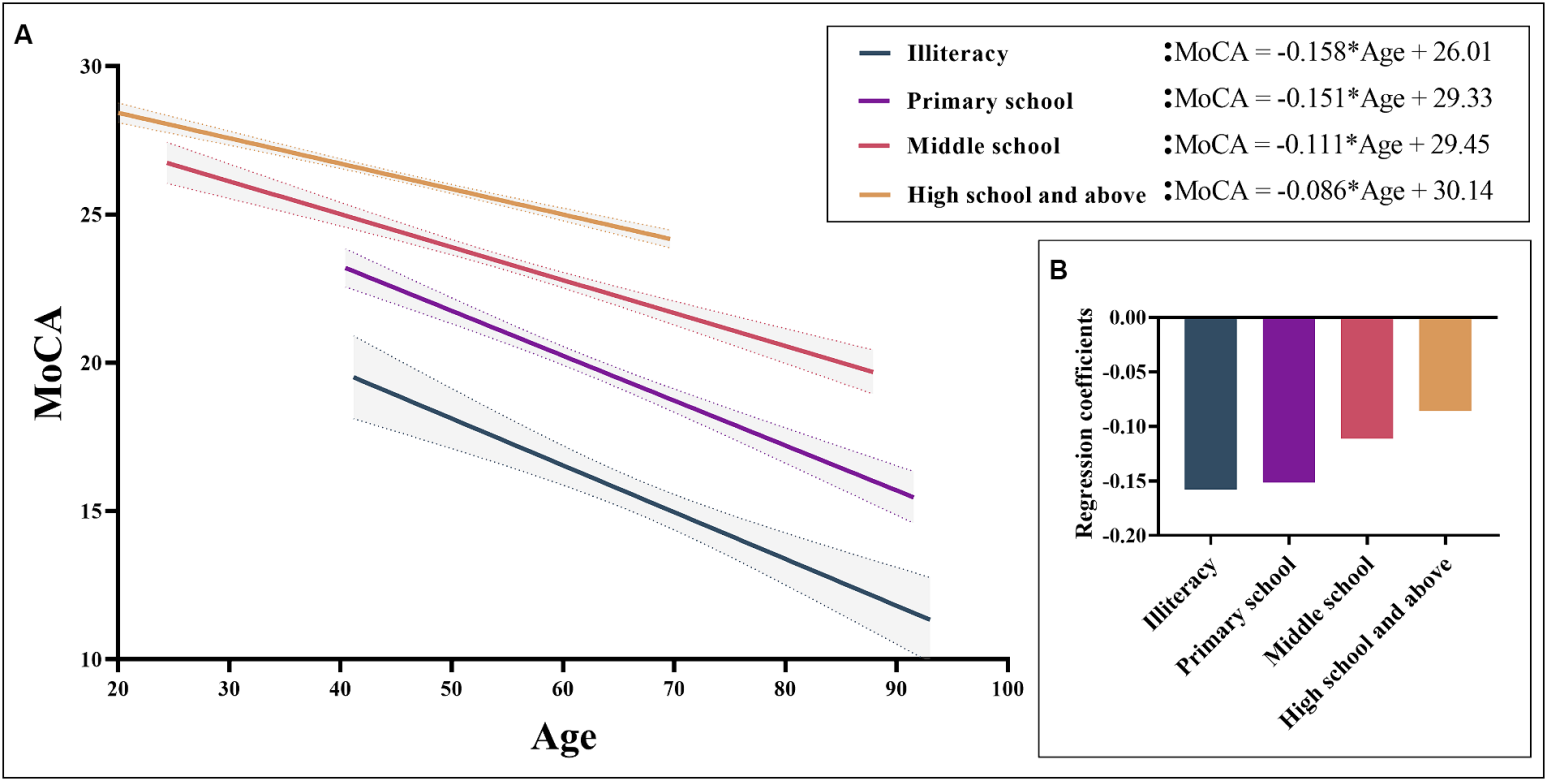
MoCA total scores based on age for different levels of education. Figure **(A)** shows a negative correlation between MoCA total scores and age can be observed in different educational levels. Figure **(B)** shows the regression coefficients of different groups, showing that the lower the education level, the faster the cognitive decline with age. MoCA, Montreal Cognitive Assessment.

Normative data stratified by age and education were derived. The mean MoCA score of the participants was 23.25 (SD = 4.82) and was higher among the youngest participants and those with the highest education. Due to the popularization of education, no participants under the age of 40 with an education level of illiterate or elementary school were recruited. Normative data stratified by age and education can be seen in Table 3. As education level decreases, MoCA total score decrease faster with age, as shown in Figure 2.

**Table 3:** MoCA total score by age and education level.

Age group, y	Education level																			
	Illiteracy				Primary school				Middle school				High school and above				Total by age			
	N	Mean	SD	Median	N	Mean	SD	Median	N	Mean	SD	Median	N	Mean	SD	Median	N	Mean	SD	Median
<40	–	–	–	–	–	–	–	–	53	24.57	3.34	25	359	27.52	2.24	28	412	27.14	2.6	28
40–49	24	18.29	3.01	18	96	22.75	3.6	23	224	24.61	3.15	25	297	26.38	2.55	27	641	24.92	3.48	26
50–59	29	18.03	3.71	19	237	21.13	4.05	21	348	23.74	3.33	24	357	25.66	2.76	26	971	23.64	3.89	24
60–69	59	16.22	4.53	16	150	19.16	4.14	19	146	21.9	4.07	22	215	24.59	3.34	25	570	21.6	4.79	22
70–79	68	13.81	4.29	13	117	18.61	4.25	19	74	21.24	4.43	21	95	23.65	3.48	24	354	19.59	5.34	20
≥80	28	12.93	4.48	12	61	16.51	4.76	17	30	19.77	4.43	19.5	30	22.93	3.78	24	149	17.79	5.55	18
Total by education	208	15.48	4.58	15	661	20.04	4.48	20	875	23.36	3.79	24	1,353	25.94	3.04	26	3,097	23.25	4.82	24

y, Years; N, Number; SD, Standard deviation.

### Demographics and neuropsychological tests for controls and VCI

A total of 242 VCI patients and 137 age, education, and gender matched control groups were included. Detailed demographic information, MoCA total scores, and multidimensional standardized neuropsychological tests can be found in Table 4.

**Table 4:** Demographics and neuropsychological tests for Controls and VCI.

Index	VCI (*n* = 242)	Controls (*n* = 137)	χ^2^/Z	*p* value
Demographics				
Age (years)	59.85 ± 9.20	60.82 ± 8.97	−1.019	0.308
Education (years)	6.82 ± 3.57	10.66 ± 3.38	−9.267	<0.001^***^
Sex (M: F)	147: 95	85: 52	0.062	0.803
Cognitive screening test				
MoCA total score	17.74 ± 4.33	24.15 ± 2.37	−13.810	<0.001^***^
**Standardized neuropsychological tests**				
Memory				
AVLT-immediate recall	5.52 ± 3.05	9.62 ± 2.55	−11.057	<0.001^***^
AVLT-delayed recall	5.19 ± 3.16	9.24 ± 2.65	−10.637	<0.001^***^
AVLT-recognition	11.76 ± 3.03	13.69 ± 1.35	−6.448	<0.001
Language				
VFT-1	13.27 ± 4.57	17.66 ± 3.62	−8.845	<0.001^***^
VFT-2	12.20 ± 4.43	17.71 ± 3.77	−10.505	<0.001^***^
Attention				
DS-forward	6.49 ± 1.58	7.24 ± 1.39	−4.365	0.011^*^
CTT-1	105.93 ± 50.24	61.81 ± 22.40	−9.263	<0.001^***^
Executive function				
DS-backward	3.35 ± 1.16	4.58 ± 1.32	−8.434	<0.001^***^
CTT-2	203.75 ± 94.16	116.35 ± 46.12	−10.174	<0.001^***^
Visuospatial ability				
CDT	2.88 ± 0.92	3.71 ± 0.50	−8.867	<0.001^***^

^*^Represents a significance level less than 0.05.^**^Represents a significance level less than 0.01.^***^Represents a significance level less than 0.001.VCI, vascular cognitive impairment; MoCA, Montreal Cognitive Assessment; AVLT, auditory verbal learning test; VFT-1/2, verbal fluency test (1-fruits and vegetables. 2-animals); DS, digit span; CTT, color trails test; CDT, clock drawing test.

### Cut-off value for detecting VCI

**Figure 3:**
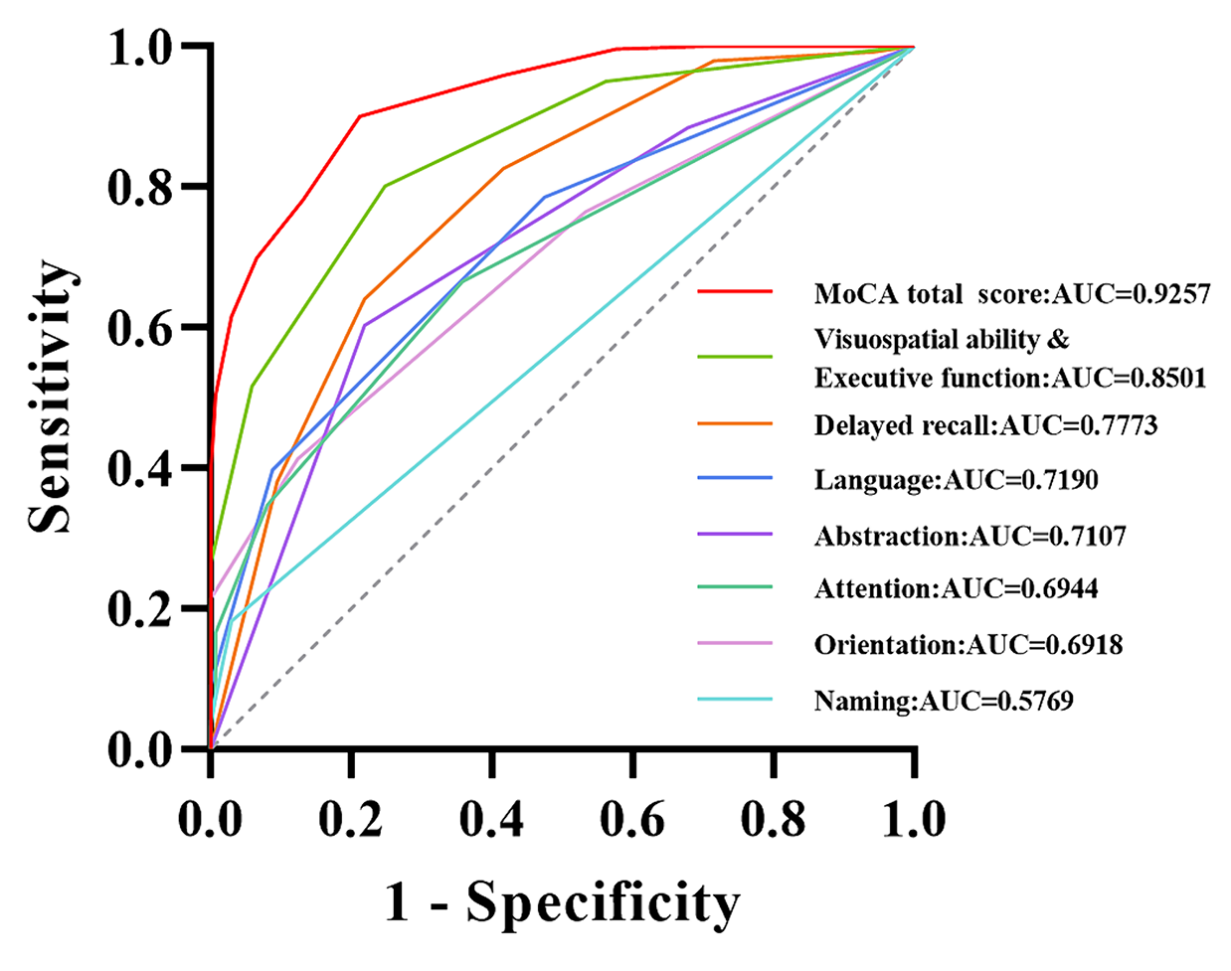
ROC curve of MoCA total score and subdomain scores for detecting VCI. The curves of different colors, respectively, demonstrate the ability of MoCA total score and subdomain scores for detecting VCI. MoCA, Montreal Cognitive Assessment; VCI, vascular cognitive impairment; AUC, area under the curve.

ROC analysis was used to evaluate the screening accuracy of MoCA total score and subdomain scores in detecting VCI and controls. The MoCA total score has excellent screening ability for detecting VCI, with an AUC of 0.9257 (95% confidence interval = 0.9007–0.9507). The optimal cut-off value for detecting VCI using MoCA total score and subdomain scores was selected and summarized in Table 5. The detailed ROC curve can be seen in Figure 3. In addition, we also summarized the screening ability of MoCA total score and the combinations of subdomains for detecting VCI in Supplementary Table S1 and Supplementary Figure S2.

**Table 5:** The performance of MoCA total score and subdomain scores in detecting VCI.

Diagnosis	Cut-off	AUC	Sensitivity	Specificity	Younden’s Index
MoCA total score	≤22	0.9257	0.9008	0.7883	0.6891
Visuospatial ability & Executive function	≤3	0.8501	0.8017	0.7518	0.5535
Naming	≤2	0.5769	0.1818	0.9708	0.1526
Attention	≤5	0.6944	0.6653	0.6423	0.3076
Language	≤2	0.719	0.7851	0.5255	0.3106
Abstraction	<1	0.7107	0.6033	0.7810	0.3843
Delayed recall	≤1	0.7773	0.6405	0.7810	0.4215
Orientation	≤4	0.6918	0.4132	0.8759	0.2891

MoCA, Montreal Cognitive Assessment; VCI, vascular cognitive impairment; AUC, area under the curve.

## Discussion

This study presents normative MoCA (Mandarin-8.1 version) data stratified by age and education for individuals in Mainland China. The data were collected from a large, population-based sample, including healthy adults from 22 provinces, autonomous regions, and municipalities, thus providing a good representation of the Chinese population to a certain extent. The average age of the participants in this study was 54.46 years old (SD = 14.38), with an average education period of 9.49 years (SD = 4.61). The average MoCA score of the study population was 23.25 points (SD = 4.82). Table 3 comprehensively presents the MoCA norm data based on age and education level. Meanwhile, this study further indicates that MoCA total score is mainly influenced by age and education level, and the relationship with gender is not significant. In addition, this study found that a score of 22 was the optimal threshold for detecting VCI, with high sensitivity, specificity, and AUC value.

It is widely recognized that demographic factors such as age and educational attainment exert a substantial influence on cognitive function. In this study, multiple linear regression analysis demonstrated that MoCA scores are predominantly influenced by years of education and age, while no statistically significant relationship was observed with gender. These results are consistent with previous studies on MoCA, which have sparked considerable debate over the potential impact of gender on MoCA total score (Freitas et al., 2011; Lu et al., 2011; Kenny et al., 2013; Narazaki et al., 2013; Conti et al., 2015; Malek-Ahmadi et al., 2015; Kopecek et al., 2017; Rossetti et al., 2017). Age and education accounted for 46.8% of the variance in MoCA raw scores, a proportion that is notably higher than the normative data reported in recent years in some other countries (Borland et al., 2017; Thomann et al., 2018; Classon et al., 2022; Gagnon et al., 2022). This could be attributed to our substantial sample size and comprehensive representation of various age groups and education levels (Thomann et al., 2018). This study aligns with the findings of previous research, as it indicates that younger age and higher education levels are associated with higher MoCA scores (Rossetti et al., 2011; Kenny et al., 2013; Narazaki et al., 2013; Santangelo et al., 2015; Tan et al., 2015; Konstantopoulos et al., 2016; Larouche et al., 2016; Mellor et al., 2016; Borland et al., 2017; Kopecek et al., 2017). Hence, when assessing cognitive impairment using MoCA, it is imperative to take into comprehensive account the subjects’ age and educational background. Across the four distinct educational strata examined in this study, a discernible pattern emerged wherein MoCA scores exhibited a reduction in tandem with advancing age. Moreover, our investigation revealed a noteworthy observation: individuals possessing higher educational qualifications demonstrated comparatively attenuated cognitive decline in relation to age. This overarching trend is graphically depicted in Figure 2. This aligns coherently with prior research outcomes that have similarly underscored the positive correlation between higher education levels and superior cognitive function. Essentially, education serves as a protective factor for cognitive function (Zhu et al., 2017; Kim, 2020; Liu et al., 2021; Rahe and Quaiser-Pohl, 2023).

In earlier work Nasreddine et al. (2005), previously set a score of 26 as the cut-off value for cognitive impairment, a benchmark that has garnered widespread recognition among researchers for an extended period of time. However, it is worth noting that there is still considerable doubt as to whether 26 points are suitable as a cut-off value for cognitive impairment in different countries or ethnic groups. This did not surpass our anticipated psychological expectations, as this has been mentioned in previous studies focused on normative MoCA data across diverse countries. And these investigations have revealed that relatively few participants attain a score of 26, and a substantial proportion of participants exhibit younger ages or possess higher education levels (Freitas et al., 2011; Borland et al., 2017; Kopecek et al., 2017; Rossetti et al., 2017; Davis et al., 2021). Our study further supports this viewpoint from the perspective of the Chinese population and revealed that the previously established cut-off value of 26 points may not be suitable for the Chinese population. For among the 3,097 participants in this study, the overall mean was 23.25, merely 1,204 (38.88%) achieved a score equal to or higher than the conventional cut-off points of 26, as proposed by Nasreddine et al. (2005). Meanwhile, a recent study demonstrated that although the critical value of 26 points exhibits high sensitivity in detecting dementia, its specificity is comparatively suboptimal (Davis et al., 2021). Therefore, it is even more important for the Chinese population to develop more realistic MoCA standardized values. It is important to highlight that the study conducted by Nasreddine et al. (2005) differs significantly from our research in terms of research design and objectives. Their study primarily aimed to ascertain the most effective score for distinguishing between healthy volunteers, individuals with mild cognitive impairment, and dementia patients, with a relatively higher educational level among the subjects (Engedal et al., 2022). In contrast, our research focused on generating normative MoCA data applicable to diverse age groups and education levels among the Chinese population. As illustrated in Table 1, this study encompasses potential reasons for the substantial variance observed in the 26-point cut-off when compared to our research findings. The MoCA scale is composed of multiple subdomain tests, representing various dimensions of cognition. In this study, we explored the efficacy of individual subdomain scores and combined subdomain scores in detecting VCI. As shown in Table 5 and Figure 3, the screening ability of single subdomain scores for detecting VCI is relatively insufficient. Notably, Supplementary materials indicate that certain combinations of subdomains exhibit promising AUC, sensitivity, and specificity, particularly the combination of visuospatial ability & executive function with delayed recall. Additionally, the screening efficiency improves with the inclusion of more subdomains. These findings align well with the characteristics of vascular cognitive impairment (Badji et al., 2023). However, it is clear that the MoCA total score remains the optimal choice for screening.

There are currently many types of threshold values for the detecting VCI, which has caused great trouble (Koski, 2013; Salvadori et al., 2013; Tu et al., 2013; Chiti and Pantoni, 2014). It should be noted that due to the complex etiology of VCI, it may even belong to different stages. Although high threshold values can achieve high sensitivity, their specificity is relatively low (Nasreddine et al., 2005), resulting in poor clinical practicality. This study determined the detecting VCI by integrating the results of multidimensional neuropsychological tests. Subtracting 1.5SD below the mean is defined as a more stable result and a false positive diagnosis of farmland. Our research results show that the cut-off value (≤ 22 points) of the Mandarin 8.1 version of the MoCA scale for detecting VCI has a high AUC. This is consistent with the results of previous related studies, which have also found that a cutoff value of 22/23 is more reasonable, and their sensitivity and specificity are similar to those of this study (Lees et al., 2014; Potocnik et al., 2020).

It must be mentioned that this study also has some limitations. Firstly, despite the wide geographical distribution of participants, there exist substantial variations in the number of enrolled individuals across different regions. Secondly, even if there are no obvious complaints of cognitive impairment, we did not screen for too many potential factors that may affect cognitive function (excluding stroke) and did not use strict cognitive assessments to evaluate participants’ cognitive function. Therefore, it remains possible that latent cognitive impairments among the subjects cannot be entirely ruled out. Thirdly, we did not employ MMSE to further evaluate the consistency of the revised MoCA, a choice that also partially mitigated the potential bias stemming from the learning effects associated with administering analogous tests.

## Conclusion

This study provides MoCA with normative values based on age and education years for the Chinese population. Our research findings further confirm the contribution of age and years of education to MoCA total score, and further indicate that the cut-off value of 26 points is not suitable for the Chinese population. Moreover, for patients with vascular cognitive impairment, a score of ≤22 may be a better cut-off value.

## Data availability statement

The datasets presented in this study can be found in online repositories. The names of the repository/repositories and accession number(s) can be found at: The anonymous raw data of the normative data in this study has been publicly available on Science Data Bank (https://doi.org/10.57760/sciencedb.10751) and can be obtained under the Creative Commons Attribution-Non-Commercial 4.0 International License (CC BY-NC 4.0).

## Ethics statement

The studies involving humans were approved by Anhui Medical University Ethics Committee. The studies were conducted in accordance with the local legislation and institutional requirements. The participants provided their written informed consent to participate in this study.

## Author contributions

QW: Data curation, Funding acquisition, Methodology, Writing – original draft, Writing – review & editing. BD: Data curation, Formal analysis, Investigation, Methodology, Writing – original draft, Writing – review & editing. YL: Formal analysis, Investigation, Methodology, Writing – original draft. SC: Data curation, Formal analysis, Investigation, Methodology, Writing – original draft. SY: Formal analysis, Investigation, Methodology, Writing – original draft. YZ: Formal analysis, Investigation, Methodology, Writing – original draft. RY: Data curation, Methodology, Formal analysis, Writing – review & editing. TB: Conceptualization, Data curation, Investigation, Methodology, Writing – original draft. XW: Conceptualization, Data curation, Methodology, Writing – original draft, Investigation. YT: Conceptualization, Data curation, Methodology, Writing – original draft. PH: Conceptualization, Data curation, Formal analysis, Funding acquisition, Methodology, Writing – review & editing. KW: Conceptualization, Data curation, Formal analysis, Funding acquisition, Methodology, Writing – review & editing.

## Footnotes

^1^www.mocatest.org

## Supplementary material

SUPPLEMENTARY FIGURE S1 The contribution of demographic factors to the total score and sub domain scores of MoCA. The standard regression coefficients of age, education, and gender on the total score and subdomains of MoCA. *Represents a significant level less than 0.05, ^+^represents a significant level greater than 0.05, MoCA, Montreal Cognitive Assessment.

SUPPLEMENTARY FIGURE S2 ROC curve of MoCA total score and combination score of subdomains for detecting VCI. Combine subdomains based on AUC and gradually eliminate the subdomain with the lowest AUC. The curves of different colors respectively demonstrate the ability of total MoCA score and the combination of subdomains for detecting VCI. MoCA, Montreal Cognitive Assessment, VCI, vascular cognitive impairment, AUC, area under the curve, Combination 1: Visuospatial ability& Executive function & Delayed recall & Language & Abstraction & Attention& Orientation, Combination 2: Visuospatial ability& Executive function& Delayed recall & Language & Abstraction & Attention, Combination 3: Visuospatial ability& Executive function& Delayed recall & Language & Abstraction, Combination 4: Visuospatial ability& Executive function& Delayed recall & Language, Combination 5: Visuospatial ability& Executive function& Delayed recall.
