# Supplementary figures and images for: The Montreal cognitive assessment: normative data from a large, population-based sample of Chinese healthy adults and validation for detecting vascular cognitive impairment
Source: Front Neurosci. 2024 Jul 31;18:1455129. doi: 10.3389/fnins.2024.1455129 (PMC11322342; doi:10.3389/fnins.2024.1455129)

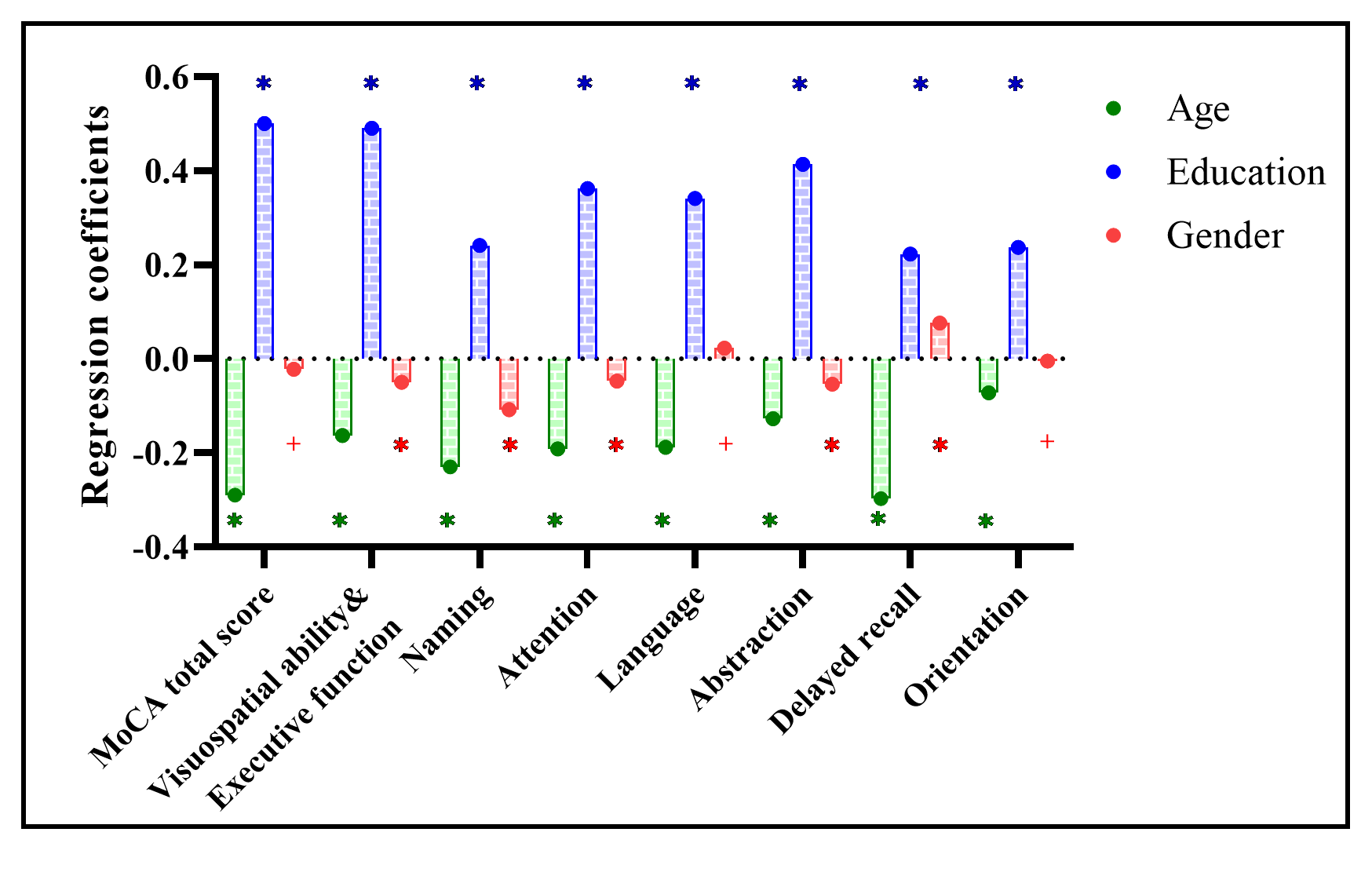

Supplement: SUPPLEMENTARY FIGURE S1 — The contribution of demographic factors to the total score and sub domain scores of MoCA. The standard regression coefficients of age, education, and gender on the total score and subdomains of MoCA. *Represents a significant level less than 0.05, +represents a significant level greater than 0.05, MoCA, Montreal Cognitive Assessment. [file Image_1.TIF]

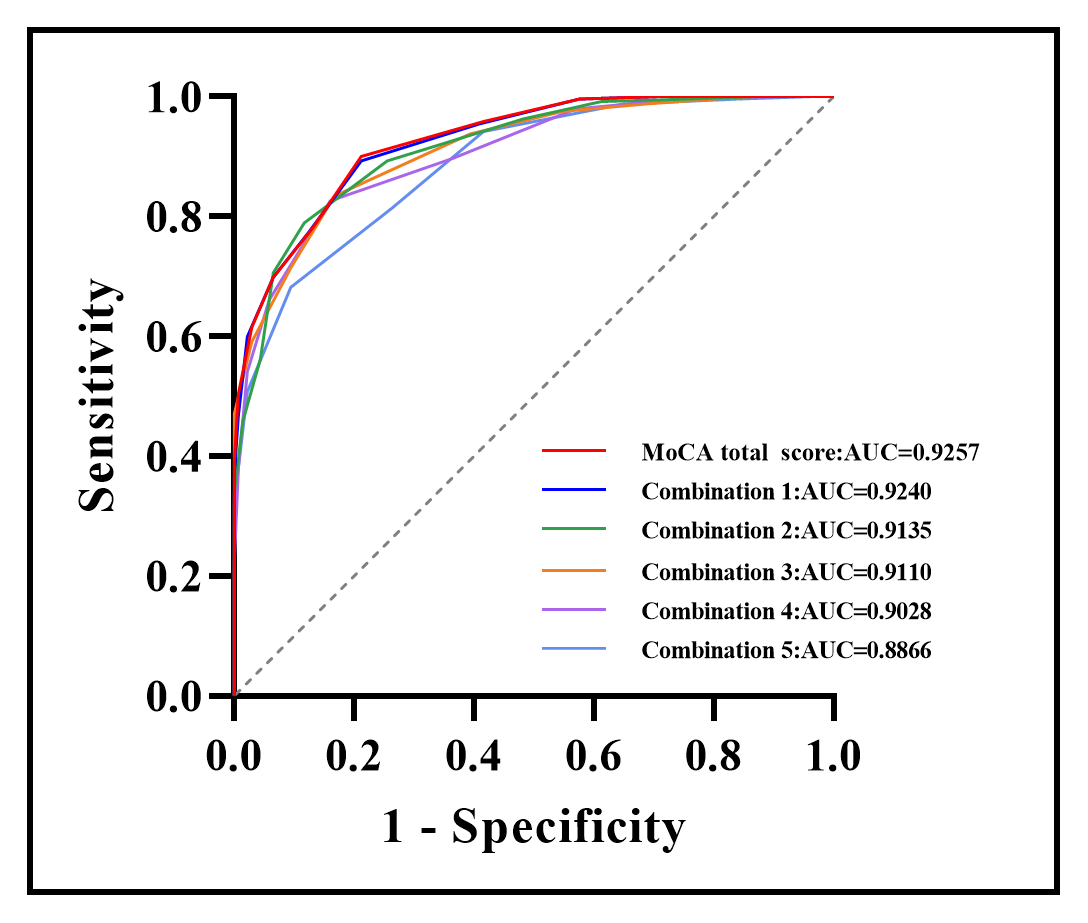

Supplement: SUPPLEMENTARY FIGURE S2 — ROC curve of MoCA total score and combination score of subdomains for detecting VCI. Combine subdomains based on AUC and gradually eliminate the subdomain with the lowest AUC. The curves of different colors respectively demonstrate the ability of total MoCA score and the combination of subdomains for detecting VCI. MoCA, Montreal Cognitive Assessment, VCI, vascular cognitive impairment, AUC, area under the curve, Combination 1: Visuospatial ability& Executive function & Delayed recall & Language & Abstraction & Attention& Orientation, Combination 2: Visuospatial ability& Executive function& Delayed recall & Language & Abstraction & Attention, Combination 3: Visuospatial ability& Executive function& Delayed recall & Language & Abstraction, Combination 4: Visuospatial ability& Executive function& Delayed recall & Language, Combination 5: Visuospatial ability& Executive function& Delayed recall. [file Image_2.TIF]
