# Supplementary material for: The Montreal cognitive assessment: normative data from a large, population-based sample of Chinese healthy adults and validation for detecting vascular cognitive impairment
Source: Front Neurosci. 2024 Jul 31;18:1455129. doi: 10.3389/fnins.2024.1455129 (PMC11322342; doi:10.3389/fnins.2024.1455129)
Supplement: Supplementary file 3 [file Table_1.docx]

**Supplementary Table 1**. The performance of MoCA total score and subdomain combination score in detecting VCI.

| Diagnosis | Cut-off | AUC | Sensitivity | Specificity | Younden’s Index |
| --- | --- | --- | --- | --- | --- |
| MoCA Total score | ≤22 | 0.9257 | 0.9008 | 0.7883 | 0.6891 |
| Combination 1 | ≤19 | 0.9240 | 0.8926 | 0.7883 | 0.6809 |
| Combination 2 | ≤13 | 0.9135 | 0.7893 | 0.8832 | 0.6725 |
| Combination 3 | ≤8 | 0.9110 | 0.8347 | 0.8321 | 0.6668 |
| Combination 4 | ≤7 | 0.9028 | 0.8264 | 0.8394 | 0.6658 |
| Combination 5 | ≤4 | 0.8866 | 0.6818 | 0.9051 | 0.5869 |

**Abbreviations:** MoCA: Montreal Cognitive Assessment, VCI: vascular cognitive impairment, AUC: area under the curve, Combination 1: Visuospatial ability& Executive function& Delayed recall & Language & Abstraction & Attention& Orientation, Combination 2: Visuospatial ability& Executive function& Delayed recall & Language & Abstraction & Attention, Combination 3: Visuospatial ability& Executive function& Delayed recall & Language & Abstraction, Combination 4: Visuospatial ability& Executive function& Delayed recall & Language, Combination 5: Visuospatial ability& Executive function& Delayed recall.
